# Drug discovery and development for Parkinson’s disease: are preclinical models good enough?

**DOI:** 10.3389/fnagi.2025.1692592

**Published:** 2025-10-28

**Authors:** Alejandro Reinares-Sebastián, Noelia Esteban-García, Masahiko Takada, Inés Trigo-Damas

**Affiliations:** ^1^HM Centro Integral de Neurociencias Abarca Campal (CINAC), Hospital Universitario HM Puerta del Sur, HM Hospitales, Madrid, Spain; ^2^Instituto de Investigación Sanitaria HM Hospitales, Madrid, Spain; ^3^CIBERNED (Center for Networked Biomedical Research on Neurodegenerative Diseases), Instituto Carlos III, Madrid, Spain; ^4^PhD Program in Neuroscience Autónoma de Madrid University–Cajal Institute, Madrid, Spain; ^5^Center for the Evolutionary Origins of Human Behavior, Kyoto University, Inuyama, Aichi, Japan; ^6^Department of Neurology, Graduate School of Medicine, Osaka University, Osaka, Japan; ^7^Centro Universitario HM Hospitales de Ciencias de la Salud (CUHMED), Universidad Camilo José Cela, Madrid, Spain

**Keywords:** Parkinson’s disease, models, drug development, motor symptoms, non-motor symptoms, preclinical trials, therapies

## Abstract

Parkinson’s disease (PD) remains a major challenge for translational neuroscience, with an increasing global prevalence and persistent unmet therapeutic needs. While its classical motor symptoms, such as bradykinesia, rigidity, and tremor, are well characterized, the clinical spectrum extends to diverse and often disabling non-motor manifestations, including hyposmia, constipation, and sleep disturbances. These features typically precede motor deficits and may dominate the late stages of disease. Despite decades of research, existing treatments remain primarily symptomatic and fail to halt disease progression. This situation has driven the development of a broad repertoire of preclinical models—ranging from *in vitro* cellular systems to complex animal models—to better understand pathogenesis and identify disease-modifying strategies. However, significant translational gaps persist, partly due to limitations in how well these models recapitulate the heterogeneity and complexity of human PD. In this review, we critically examine the main preclinical models available for PD, assessing their strengths and weaknesses for modeling both motor and non-motor features. We discuss recent advances, persistent challenges, and highlight key considerations for improving the predictive value of experimental models in drug discovery for Parkinson’s disease.

## Introduction

Parkinson’s disease (PD) is the second most common neurodegenerative disease after Alzheimer’s disease (Reichmann et al., 2009). The number of people affected by PD has been estimated at 70 million and with the progressive aging of the population over the next 10 years the number could double. In Europe alone, it represents 14 billion euro in direct and indirect costs and this amount is only expected to gradually increase (Bach et al., 2011; Chaudhuri et al., 2024). In most cases, PD is a sporadic idiopathic disease that could be related to environmental factors, and only 10–15% of cases could be related to familiar PD such as *SNCA*, *LRRK2*, *VPS35*, *PRKN*, *PINK*1, *GBA*, and *DJ-1* mutations (Blesa et al., 2016a,b; Shadrina and Slominsky, 2021).

The cardinal motor symptoms of PD are mainly associated with the degeneration of dopaminergic neurons, starting in the ventrolateral region of the substantia nigra pars compacta (SNc) (Blesa et al., 2022). When the loss of nigrostriatal dopaminergic neurons reaches 50% and the striatal dopamine (DA) deficit increased up to 80%, cardinal motor symptoms (akinesia, rigidity, and tremor) appear (Marsden, 1990; Fearnley and Lees, 1991; Armstrong and Okun, 2020). The development of the first motor symptoms is preceded by a series of non-motor symptoms (hyposmia, alterations of the rapid eye movement (REM) phase, sleep behavior disorders (RBD) and constipation) (Postuma and Berg, 2016; Schapira et al., 2017; Berg et al., 2018; Bloem et al., 2021). Interestingly, these symptoms may end up dominating the clinical profile in the last years of the disease (Blesa et al., 2022). The duration of the preclinical phase is unknown; however, it could be extended from 10 to 15 years before diagnosis (Blesa et al., 2017; Tolosa et al., 2021). Another neuropathological hallmark of PD is the presence of Lewy bodies (LB) in the dopaminergic neurons, whose main component is the *α*-synuclein (α-syn) protein. The accumulation of α-syn becomes widespread in the brain during the progression of PD (Braak et al., 2003). Although it is considered a hallmark, the presence of α-syn protein may also occur in the natural aging process of the brain and in other synucleinopathies (Greffard et al., 2010; Shahmoradian et al., 2019).

Traditionally, pharmacological treatment of PD is symptomatic and based on L-DOPA because DA or other exogenous catecholamines cannot cross the blood–brain barrier (BBB) (Rautio et al., 2008; Lafuente et al., 2019). However, chronic treatment with L-DOPA causes motor complications (on–off phenomenon) in most patients, associated with abnormal involuntary movements (dyskinesia) (Ahlskog and Muenter, 2001; Porras et al., 2015). Despite the difficulties in PD treatment, there are currently 136 agents in different trial phases and therapeutic categories according to the study by McFarthing et al. (2024), and this number has not greatly varied throughout recent years. Although only a few drugs have reached clinical trials for proving their effectiveness, none of them have been shown to fundamentally slow the pathology (McFarthing et al., 2022, 2024).

The clinical need for new PD-modifying strategies has led to the development of a wide variety of experimental models, from *in vitro* to animal models, and focused on improving pathogenic understanding of PD and validating potential therapies (Blesa et al., 2016a,b; Chia et al., 2020; Balzano et al., 2023). The translatability of experiments is based on the correct choice of experimental models that can simulate human pathology. This means that they must have features of human PD, theoretical rationales, and responses to treatments comparable to the PD patients (Blesa and Przedborski, 2014; Kin et al., 2019). However, most drug candidates that seem promising in the preclinical phases fail in clinical trials, making clear the gap in the transformation of preclinical to clinical outcomes (Del Rey N. L.-G. et al., 2018; Del Rey N. L. et al., 2018; Trigo-Damas et al., 2018). This manuscript aims to review the strengths and limitations of different experimental models available for PD and discuss their usefulness in drug discovery and development.

## Preclinical platforms: from yeast, cells, and organoids to animals

In the last 20 years, yeast, mainly *Saccharomyces cerevisiae*, have been extensively used for the development of experimental models for neurodegenerative diseases. In essence, this type of model is based on two strategies: (1) study of heterologous expression of genes of monogenic diseases; (2) analysis of the functional activity of pathologically significant human orthologous genes (Dunham and Fowler, 2013). In the case of PD, both strategies have been used indistinctly, however, the first strategy is worth highlighting since it has been used for the analysis of the genes that encode the proteins parkin, PINK1, LRRK2, and *α*-syn (this last protein being the most studied in this models) (Dunham and Fowler, 2013; Shadrina and Slominsky, 2021).

**Figure 1:**
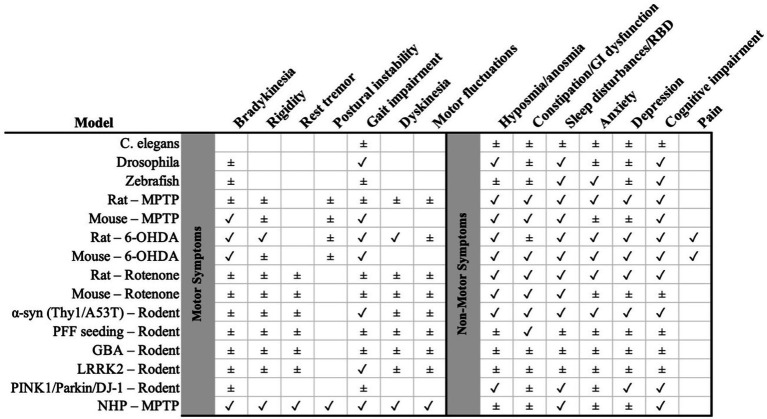
Summary matrix of preclinical Parkinson’s disease models (rows) versus clinical features (columns), with motor symptoms on the left and non-motor symptoms on the right (separated by the gray divider). Symbols indicate the level of support: ✓ consistent evidence that the feature is reproduced by the model; ± partial/heterogeneous or indirect evidence. NHP, non-human primate; α-syn, alpha-synuclein; PFF, pre-formed fibrils; 6-OHDA, 6-hydroxydopamine; MPTP, 1-methyl-4-phenyl-1,2,3,6-tetrahydropyridine; GBA, glucocerebrosidase; LRRK2, leucine-rich repeat kinase 2; PINK1/Parkin/DJ-1 as indicated. GI, gastrointestinal; RBD, REM sleep behavior disorder.

Definitely, yeast models have allowed to expand our knowledge on the toxicity of α-syn inclusions (Auluck et al., 2010; Büttner et al., 2013). One of the most remarkable result obtained in this models have been the use of expression of genes encoding heat shock proteins to reduce α-syn toxicity; while deletion of genes encoding chaperones increased its toxicity (Chen et al., 2005; Bandres-Ciga et al., 2019; Billingsley et al., 2019). Thus, these types of models have opened the door to their use as a powerful screening tool to identify and optimize new compounds or drugs that reduce α-syn aggregation, such us flavonoids (Griffioen et al., 2006; Kritzer et al., 2009), which have been tested in rats and mice with promising results, reducing muscle tremor, reversed behavior deficits, striatal dopamine depletion, and neuronal cell loss (Figure 1; Mu et al., 2016; Ay et al., 2017; de Andrade Teles et al., 2018).

Despite yeast models having been the first approximation to shedding a light on metabolic pathways and /or proteins, these models should not be considered as ideal platforms for clinical trials due to their natural limitations, as a simplified model system and, therefore, need deeper confirmation in more complex models (Surguchov, 2021).

Cell models, due to their easy handling, rapid growth and simplicity usually become the first approach for modeling neurodegenerative diseases such as PD. Some examples are still being used in laboratories as the neuroblastoma cell line (SH-SY5Y), the pheochromocytoma cell line (PC12), the Neuro-2a cell line or the MN9D cell line (Falkenburger et al., 2016; Lázaro et al., 2017). At the same time, it is worth mentioning the human mesencephalic cells (LUHMES), that have a stable dopaminergic phenotype even with similar electrical properties (Falkenburger et al., 2016; Lázaro et al., 2017).

Cell models provide opportunities to develop pharmacologic or genetic studies in a relatively simple way, offering strategies to modulate the disease pathways. Noteworthy, one relevant example of the potential of these models is exenatide, a glucagon-like peptide-1 (GLP-1) receptor agonist resistant to enzymatic degradation, which demonstrated neuroprotective effects *in vitro*. It was tested in rat PC12 cells, which possess synthesis, metabolism and transporter systems of dopamine, and it was capable of inducing neurite growth and promoting neuronal differentiation. Following its promising results in preclinical PD models, the drug has advanced to phase III clinical trial (NCT04232969) (see Supplementary Table 1; Foltynie and Aviles-Olmos, 2014; Athauda et al., 2017; Charvin et al., 2018).

Interestingly, the search for personalized treatments has made the use of other cell culture approaches of growing relevance. An example of this is induced pluripotent stem cells (iPSCs), which can be reprogrammed from patient-derived somatic cells and differentiated into dopaminergic neurons (Torrent et al., 2015). A pioneering case in the USA demonstrated that the autologous transplantation of iPSC-derived dopaminergic progenitors, resulted in graft survival, functional evidence of dopamine production, and clinical improvement during follow-up (Figure 1; Schweitzer et al., 2020). In parallel, the phase 1/2 Kyoto trial (jRCT2090220384) (see Supplementary Table 1), initiated in 2018, employed cryopreserved allogeneic iPSC-derived dopaminergic progenitors with varying degrees of HLA compatibility to evaluate the safety and efficacy of this approach in PD patients (Takahashi, 2020). Although these strategies confirm the feasibility of iPSC-bases therapies, challenges remain regarding their high cost, complex production processes and, the need for immunosuppression. As an alternative, dopaminergic progenitors derived from human embryonic stem cells (hESCs) have been explored as an allogenic source. The first clinical trial with hESCs, reported by Wang et al., 2017 (NCT083119636) (see Supplementary Table 1), demonstrated the feasibility of such transplants in PD patients (Wang et al., 2018). Subsequently, two additional phase 1 clinical trials (NCT04802733 and STEM-PD) (see Supplementary Table 1) were initiated, confirming the safety and functional integration of the grafts in patients with PD (Kirkeby et al., 2017; Tabar et al., 2025).

In addition, CRISPR/cas9 has emerged as a novel technique in cell cultures and it has expanded the possibility of genome editing (Soldner et al., 2016). In summary, these two approaches provide closer alignment with the characteristics of PD patients, thereby facilitating studies designed to evaluate personalized treatments or compounds. However, they have not been able to establish pathological phenotypes of PD, since these are based on both mutations and environmental factors. One solution to this is the use of organoids 3D, allowing to investigate the genotype–phenotype relationships in PD, maintaining translational aspects of the disease (Jo et al., 2021). Organoid methodology allows the development of more accurate human cellular models for the investigation of neurodegenerative disorders, where biological processes- such as cell–cell interactions, pathological cascades, or spatial organization- that are not adequately reproduced in two-dimensional cultures (Smits et al., 2019). Furthermore, by imitating physiological conditions it allows testing new drugs or evaluating existing ones (Zhang et al., 2024; Frattini et al., 2025) Additionally, it provides a realistic reproduction of the blood brain barrier (BBB), which is crucial for advancing treatment strategies for PD (Cui et al., 2024; Dao et al., 2024). In conclusion, 3D cell culture systems serve as a bridge between traditional *in vitro* experiments and *in vivo* animal models (Smits and Schwamborn, 2020).

Nevertheless, this model has inherent limitations, and data derived from cell lines provide an important contribution to research by validating and refining the interpretation of results when applied alongside more advanced animal models such as the *Drosophila*, murine or other mammal model.

### Modeling motor symptoms of Parkinson’s disease

Experimental models for PD have been pivotal in mimicking the hallmark motor symptoms—bradykinesia, rigidity, and tremor—providing platforms for understanding disease mechanisms and testing therapeutic approaches (He et al., 2024). These models include invertebrate organisms, and vertebrate animals, each contributing uniquely to the translational pipeline. These models—ranging from neurotoxin-induced lesions in rodents and primates to genetically engineered animals—aim to replicate key aspects of the disease, particularly its motor manifestations (Dovonou et al., 2023). While no single model fully recapitulates all facets of PD, each provides valuable insights into specific disease mechanisms and treatment responses (Barker and Björklund, 2020). The use of such models has been pivotal in elucidating dopaminergic pathways, evaluating pharmacological interventions like levodopa and dopamine agonists, and exploring novel approaches including gene and cell therapies (Chen et al., 2023).

#### Invertebrate models

These organisms share many conserved molecular pathways and cellular processes with humans and have been successfully used to study environmental and genetic factors (Fatima et al., 2018). Their rapid growth, low-cost maintenance, easy manipulation and well-known genome have made them a useful and ethically less problematic pharmacological screening tool compared to other animal models (Surguchov, 2021). Unfortunately, the absence of a complex physiological network, and the lack of the orthologous *α*-syn gene in most invertebrate models, have shown differences in both the pathogenic pathway and the effects on it. These disparities among models may arise from inherent variations between species or differences in the methodologies employed (Shadrina and Slominsky, 2021; Surguchov, 2021).

##### Caenorhabditis elegans

*C. elegans* is a microscopic nematode characterized by a short shelf life, many progeny and easy cultivation. This nematode has a small and well-studied genome with many orthologous PD-related genes, however it lacks the genes that encode for α-syn protein in humans (Shaye and Greenwald, 2011; Cooper and Van Raamsdonk, 2018).

###### Caenorhabditis elegans—toxic-based

This model allows the use of different neurotoxins including 6-hydroxydopamine (6-OHDA), paraquat, rotenone, and 1-methyl-4-phenylpyridinium (MPP+), resulting in selective neurodegeneration of dopaminergic neurons (around 30%). Similarly, the combination of genetic models with environmental neurotoxicity exposure led to an 85% of death (Zeng et al., 2018). Accordingly, this model has been used to test novel factors with the potential of reducing the neuronal toxicity of α-syn in dopaminergic neurons and epigenetic factors (Gaeta et al., 2019; Goya et al., 2020) and to evaluate the “prion-like” α-syn hypothesis owing to its transparency which allows the visualization of neuron-to-neuron transfer of α-syn (Sandhof et al., 2020).

###### Caenorhabditis elegan—genetic

Its transparency and easy of genetic manipulation have allowed genes to be silenced in specific neuronal subtypes- such as GABAergic, serotonergic, dopaminergic, cholinergic or glutamatergic neurons- using interfering RNA (RNAi), a method that is stable during at least 3 generations (Cooper and Van Raamsdonk, 2018). One example of this, is the work by Sohrabi et al., where they screened 50 drugs approved by the Food and Drugs Administration (FDA) in the *C. elegans* neuronal bcat-1 knockdown, which causes abnormal behavior with age (Figure 1; Sohrabi et al., 2021).

##### Lymnaea stagnalis

Moreover, *L. stagnalis,* offers a relatively long lifespan and high conservation of aging-related molecules (Fodor et al., 2020). Classic studies have shown that intermittent exposure of this snail to rotenone induced significant reduction of locomotion, feeding and tyrosine hydroxylase (TH) expression in the central nervous system, suggesting that this model could be suitable for PD research (Vehovszky et al., 2007; Maasz et al., 2017; Zeng et al., 2018). The potential benefit of this model in testing new drugs and compounds becomes evident with the compound Carnosine, a natural endogenous dipeptide that has shown benefits in clinical trials (Rivi et al., 2024; O’Toole et al., 2025).

##### Drosophila melanogaster

*D. melanogaster* or fruit fly is another powerful invertebrate model that shares approximately 75% of human diseases-related genes (Marsh and Thompson, 2006). Just like the rest of the invertebrate models, the fruit fly has numerous advantages related to handling: short life cycle, large progeny, a well-known small genome that allows edition and easy manipulation, as well as the conservation of brain structures and metabolic pathways (Aryal and Lee, 2019).

###### Drosophila melanogaster—toxic-based

Toxins can be used for modeling in *Drosophila*; however, these toxic models have not been widely adopted due to difficulties in estimation of the optimal dose to obtain comparable models. Nevertheless, some studies have been successful in reproducing the main characteristics of PD in toxic models (Figure 1; Coulom and Birman, 2004; Abolaji et al., 2018; Farombi et al., 2018).

###### Drosophila melanogaster—genetic

*Drosophila*’s nervous system is not completely homologous to the human, and one example is the case of the gene encoding *α*-syn in humans which has not been found in the *Drosophila*. Thus, the characteristics associated with α-syn are usually promoted by the inclusion of *SNCA* human gene in transgenic (TG) flies (Shadrina and Slominsky, 2021). TG-flies overexpressing α-syn display a progressive loss of dopaminergic neurons, α-syn inclusion formation, and loss of climbing ability. This has allowed to differentiate the presymptomatic and symptomatic phases characteristic of PD (Xiong and Yu, 2018). In addition, there are some drugs tested in the genetic *Drosophila* model that have managed to reach clinical trials, this is the case of Resveratrol (Adedara et al., 2022), Curcumin (Siddique et al., 2014) or Pomalidomide (Palmas et al., 2022). In the preclinical study of the Pomalidomide, using the *LRRK2* genetic model, treated fruit flies managed to show improved climbing activity (Figure 1; Casu et al., 2020).

Although invertebrate models do not recapitulate the full anatomical and physiological complexity of the human nervous system, they are a powerful strategy for studying fundamental disease mechanisms, testing gene–environment interactions, and conducting large-scale drug screening. Their value resides in their capacity to yield robust and reproducible results during early-stage research, providing a foundation for validation in more complex models.

#### Vertebrate models

Vertebrate models are crucial in PD research, as they closely replicate key anatomical and physiological features of the human brain. This similarity allows us to study drug efficacy and delivery under conditions that more accurately reflect the human disease. However, this type of organisms is generally less recommended for drugs and genes screenings due to their long-life span and their complex genome, compared to the invertebrate models. Furthermore, these models can be expensive to maintain, and there are higher ethical restrictions, which limits the number and severity of the experimental procedures. These models encompass a wide phylogenetic spectrum, ranging from fish to primates, each with distinct advantages and limitations that shape their translational relevance (Blesa et al., 2018).

##### Zebrafish

The Zebrafish is distinguished by its small size, a rapid breed (3 months per generation) and a relatively simple maintenance. This model possesses a dopaminergic system located in the posterior tuberculum, functionally analogous to the human dopaminergic system (Shadrina and Slominsky, 2021). Their genomic and physiological organization makes them closer to the humans’ compared with *Drosophila*, *C. elegans* or *L. stagnalis*.

###### Zebrafish—toxic-based

Zebrafish exhibit susceptibility to various specific dopamine neurotoxins that selectively target dopaminergic neurons, including 1-methyl-4-phenyl-1,2,3,6-tetrahydropyridine (MPTP), 6-OHDA, paraquat, and rotenone (Feng et al., 2014; Sarath Babu et al., 2016; Zeng et al., 2018; Zhang et al., 2017). For example, the intraperitoneal administration of MPTP in adult zebrafish resulted in reduced locomotor activity, decreased crossing frequency and swimming distance, increased freeze bouts, and extended freeze duration (Figure 1). Additionally, this treatment led to an upregulation of γ1- and γ2-synuclein expression (Sarath Babu et al., 2016; Zeng et al., 2018). Thus, this model has been used to assess the potential effect of Rasagiline (Cronin and Grealy, 2017), a selective inhibitor of the monoamine oxidase B (MAO-B), which clinical studies have demonstrated its potential neuroprotective effect in humans (NCT02789020) (see Supplementary Table 1).

###### Zebrafish—genetic

Moreover, 70% of human genes have orthologs in the zebrafish, including those which cause familiar PD (*LRRK2*, *PRKN*, *DJ1*, and *PINK1*), though there is no evidence of orthologs of *α*-syn genes (Wang et al., 2021). However, there are other orthologous genes which deletion leads to motor deficits (Figure 1) and decreased dopamine (DA) levels, such as *β*-, γ1-, and γ2-synuclein (Barnhill et al., 2020; Goldsmith and Jobin, 2012; Stewart et al., 2014; Doyle and Croll, 2022). However, restoring gene expression or overexpressing human α-syn these deficits are solved (Milanese et al., 2012) indicating that endogenous forms of synuclein in zebrafish could perform a function comparable to the human α-syn.

On the other hand, zebrafish have some shortcomings. There are less TG-models developed compared with other animals, the number of validated reagents is limited, and they have a natural capability of regenerating cell injuries that can mask the progression of neurodegeneration (Barbosa and Ninkovic, 2016; Caldwell et al., 2019; Clarke et al., 1987). Nonetheless, zebrafish offer a rapid and cost-effective system for early-stage drug screening and toxicological studies (Razali et al., 2021; Zambusi and Ninkovic, 2020).

##### Rodents

Rodents, especially mice and rats, are the most commonly used animal model in PD, being the preferred selection in 80% of all PD studies because they entail easy and well-established experimental protocols, standardized behavioral assays and a wide range of possible TG-models (Kin et al., 2019; Chia et al., 2020).

###### Rodent—toxic-based

From the beginning of the century, the use of toxic models in rodents became widespread (Blandini and Armentero, 2012; Kin et al., 2019; Shadrina and Slominsky, 2021), been the most frequently used MPTP, rotenone, 6-OHDA and paraquat (Blesa and Przedborski, 2014; Trigo-Damas et al., 2018). These models reproduce most of the key features of PD, such as dopaminergic neuronal loss and motor impairments (Figure 1), being 6-OHDA lesions-based model is the most commonly used in pharmacological research (Hernandez-Baltazar et al., 2017; Zeng et al., 2018). Alternatively, there are other less commonly used toxin-induced PD models in mice, such as reserpine (acting on presynaptic terminals), aminochrome (promoting mitochondrial dysfunction), or trichloroethylene or β-sitosterol β-d-glucoside (BSSG) (both inducing loss of DA neurons) and titanium dioxide nanoparticles (Van Kampen et al., 2015; Segura-Aguilar, 2017; Razali et al., 2021). Also some studies are focused in the role of protofibrils and fibrils, that represent a toxic component for TH neurons (Gómez-Benito et al., 2020; Izco et al., 2021; Dovonou et al., 2023).

Two prominent pharmacological examples where the 6-OHDA model has been used as a preclinical model and have made it to clinical phases: (I) the drug CVT-301, an inhaled formulation of levodopa (Lipp et al., 2016), increased plasma levels compared to oral administration (Bartus et al., 2004). This treatment was approved in 2018 for PD patients by the U. S. Food and Drug Administration (FDA) (Grosset et al., 2013; Lewitt et al., 2016; LeWitt and Fahn, 2016; Lipp et al., 2016; Isaacson et al., 2018). (II) Apomorphine has limited oral bioavailability, which has led to the development of different routes of parenteral administration. The rapid clinical response of apomorphine (7–10 min) has made intermittent subcutaneous injections a suitable therapy for “on/off” fluctuations (Borkar et al., 2017; Katzenschlager et al., 2018).

The MPTP mice model is another important toxic model used in the study of PD. This model has been used to develop drugs that target not only the dopaminergic pathways affected in PD, but also other aspects such as mitochondrial dysfunction, protein degradation, neuroinflammation, and the accumulation of oxidative species (Blesa et al., 2015; Belarbi et al., 2017; Balzano et al., 2023). In this sense, one of the drugs that has given promising results with neurotrophic and anti-inflammatory properties is exenatide. It is a peptide receptor agonist like glucagon 1 (GLP-1) and resistant to enzyme degradation. In MPTP mouse model, exenatide has been shown to attenuate toxicity, preserving neurons in the SNc combined with a decrease in activated microglia (Li et al., 2009; Belarbi et al., 2017). Also, the synthetic androstenetriol HE3286 has been tested in the murine MPTP model demonstrating a neuroprotective effect and reduction of motor impairment and neuroinflammation (Nicoletti et al., 2012). The clinical trial of HE3286 was completed in 2023 for PD (NCT05083260) (see Supplementary Table 1).

Finally, one of the most relevant approaches of the last decade is to promote neuronal survival using neurotrophic factors to help affected neurons in PD (Heiss et al., 2019; Lindholm and Saarma, 2022; Moriarty et al., 2022). Cerebral dopaminergic neurotrophic factor (CDNF) and glia cell line-derived neurotrophic factor (GDNF) have been shown to have a strong result in restoring the function and neuroprotection of midbrain DA neurons in murine neurotoxic models (6-OHDA and MPTP) (Kirik et al., 2000; Oiwa et al., 2006; Smith and Cass, 2007). As a result of these type of preclinical studies, clinical trials have been developed in 2019 and 2020 to test the safety of CDNF by brain infusion in patients with PD (NCT03295786 or NCT03775538) (see Supplementary Table 1). One of the solutions that has been launched to solve the handicap of the direct administration of GDNF in the brain, is the use of gene therapy by viral vectors, such as adenovirus, lentivirus, and adeno-associated virus (AAV) vectors (Enterría-Morales et al., 2020; Terse et al., 2021). This route of administration has been validated with respect to direct administration studies, demonstrating that AAV-GDNF administration in the SN or striatum prevent cell death in the rat 6-OHDA unilateral model (Kordower et al., 2000; Connor, 2001; Wang et al., 2002). Other alternative to the viral-vectors, it is a neurotensin-poliplex method, which achieves by transfection the overexpression of GDNF encapsulated nanoparticles in dopaminergic cells. This allows the expression of GDNF in a controlled and prolonged manner (Espadas-Alvarez et al., 2017). Furthermore, Yurek et al. have administered nanopolysine particles and a plasmid encoding GDNF to the midbrain of rats, improving the number of TH + cells and the rotational behavior of animals (Figure 1; Yurek et al., 2017). In this sense, in 2020 started a new clinical trial to evaluate the safety and the potential effect of the AAV2-GDNF delivered to the long-lasting PD putamen patients (NCT04167540) (see Supplementary Table 1).

###### Rodent—genetic

In parallel, genetic rodent models have been used for the study of personalized modifications in the genome, replicating key pathogenic axes of PD (*α*-synuclein aggregation, lysosomal and mitochondrial dysfunction). These models have consequently driven significant advances in understanding PD pathophysiological process of this disorder (Blesa and Przedborski, 2014). However, different models of α-syn overexpression and mutations of the principal genes (*A53T*, *A30P*, and *E46K*) have been created, reporting progressive motor decline, neuritic dystrophy and intraneuronal inclusions (Giasson et al., 2002; Zarranz et al., 2004; Aniszewska et al., 2022, respectively). One example of the potential therapeutic effectiveness of these models is demonstrated in studies on: B-312 active immunization (Phase 1, NCT04075318), ABBV-0805 passive immunization (Phase 1, NCT04127695), buntanetap/ANVS401 translational inhibitor (Phase 2a, NCT04524351), and UCB0599/minzasolmin (Phase 1 NCT04875962; Phase 2 NCT04658186; extension NCT05543252), together with the anti-inflammatory hypoestoxide (NCT04858074) (see Supplementary Table 1; Kim et al., 2015; Kuo et al., 2019; Nordström et al., 2021; Nimmo et al., 2022). These data illustrate the models’ value for mechanism-based pharmacology and biomarker alignment, showing significant improvements in pathological and behavioral outcomes.

TG-mice overexpressing human wild-type *α*-syn (SNCA) have been pivotal for advancing immunotherapies in PD. One example is prasinezumab (RO7046015) reducing α-syn pathology and improving motor performance in two complementary overexpression lines, both expressing human WT (not A53T/A30P) SNCA (Pagano et al., 2021; Richter et al., 2023). These convergent mechanistic and behavioral effects provided the basis for clinical translation (Pagano et al., 2021, 2024). Also using one of these models, a small molecule misfolding inhibitor (UCB0599/minzasolmin; predecessor NPT200-11) has been tested reducing α-syn pathology and improved PD-relevant endpoints (Price et al., 2018), leading to Phase 1 clinical studies (NCT05543252) (see Supplementary Table 1).

Additionally, TG-rodents carrying the LRRK2 mutation, which mimics dysfunction in nigrostriatal dopaminergic neurotransmission, exhibit variable locomotor changes (Figure 1) and increased tau expression and/or phosphorylation (Daniel and Moore, 2015; Seegobin et al., 2020). However, more recent constructs featuring LRRK2-G2019S have shown increased efficiency, causing approximately 50% neuronal loss in the substantia nigra pars compacta (SNc) in mice (Lee et al., 2010; Xiong et al., 2018). However, many knock-in and several overexpression lines do not show a notably SNc dopaminergic cell loss (Li et al., 2010; Tsika et al., 2014; West, 2017). As a result, preclinical models featuring LRRK2 have been instrumental in the development of novel drugs aimed at improving the pathogenesis of genetic PD. These efforts have subsequently led to clinical studies in patients (Jennings et al., 2022, 2023). In the same line for the TG-rodent model but different mutation, it is important to briefly mention the mutations in *parkin* and *DJ-1* that seldom show frank nigrostriatal dopaminergic cell loss at baseline, despite progressive behavioral or biochemical changes (Yamaguchi and Shen, 2007; Chandran et al., 2008), but they consistently reveal mitochondrial/oxidative stress and proteostatic disturbances (Malkus et al., 2009; Lopert and Patel, 2014). Loss of parkin impairs mitophagy and can depress mitochondrial biogenesis (Stevens et al., 2015), positioning these lines as tractable platforms to interrogate proteostatic-mitophagic pathways and antioxidant strategies (Shin et al., 2020). While single-gene transgenic models have provided substantial insights into disease mechanisms, they often fail to reproduce the full spectrum of PD pathology. In this regard, “double-hit or “multi-hit” approaches, in which genetic susceptibility (e.g., *LRRK2*, *parkin* or other mutations) is combined with an environmental exposure such as rotenone, MPTP or inflammatory microenvironment may offer a more accurate representation of the complex and heterogeneous pathophysiology of PD (Rudyk and Hayley, 2019; Dwyer et al., 2020).

In relation with these TG models, drugs regulating other pathological pathways, such as protein degradation or reactive species, are being investigated. Of note here is ambroxol, which can regulate the activity of Gcase (an enzyme involved in lysosomal function). In the study by Migdalska-Richards et al., ambroxol increased Gcase activity and reduced the level of *α*-syn in L444P TG-mice in the Gba1 gene and in overexpressing α-syn TG-mice (Migdalska-Richards et al., 2016). Their promising preclinical findings prompted the launch of different clinical trials focused on different aspects of PD (NCT02941822 and NCT02914366) (see Supplementary Table 1).

Definitely, murine models, whether toxin-induced or transgenic, have been instrumental in advancing pharmacological studies for PD, enabling some drugs to progress to clinical phases.

##### Minipigs

Minipigs have gained popularity in recent years due to their anatomical and physiological similarities with humans, particularly with regards to the brain. The large size of the SNc and striatum in minipigs make them particularly suitable for neuroimaging and electrophysiological studies. The study of PD in minipigs started to surface back in 1999 when M. Mikkelsen et al. administered MPTP to nine pigs, which caused the animals to develop muscle rigidity, hypokinesia and impaired coordination (Figure 1). Also, the histological assessment revealed a reduction of the striatal amount of DA and a decrease of the number of DA cells in the SNc, 3 months after MPTP administration (Mikkelsen et al., 1999). More recent studies have administered different toxins such as 6-OHDA in the nigrostriatal pathway, or have inoculated lactacystin in the forebrain inhibiting the ubiquitin proteasome system (Christensen et al., 2018; Lillethorup et al., 2018; Zhu et al., 2018; Del Rey et al., 2021). In the past few years, genetic models based on this animal with human *SNCA* or simultaneous mutations in different genes using CRISPR/Cas9-mediated gene editing have been created (Mehmood et al., 2021; Shadrina and Slominsky, 2021). As in murine models, drugs of new formulations of L-DOPA, such as ND0612, have been tested. ND0612 is a liquid formulation of levodopa and carbidopa that can be administered subcutaneously through a patch device. It has completed phase III trials and has shown promising results (Ramot et al., 2017; McFarthing et al., 2021). Moreover, research utilizing subcutaneous apomorphine and emerging treatments such as GDNF has been undertaken in preclinical trials employing the minipig model. These studies, highlighted by Ramot et al. (2018), Ramot et al. (2017), and Wahlberg et al. (2020), underscore the adaptability and efficacy of this model in advancing novel therapies for Parkinson’s disease. These instances illustrate merely a fraction of the potential contributions the minipig.

##### Dogs

Dogs have been categorized as a natural model of PD due to significant similarities in diet, microbiomes and neuropathological features (Ambrosini et al., 2019). Furthermore, these models have been used to test pharmacology treatments, such as the CV-301, where maximum absorption times were studied in a dog model (beagle) (Watanabe et al., 2015; Jeong et al., 2016; Lipp et al., 2016). MPTP-intoxicated dogs exhibit clinical symptoms resembling those of PD, including akinesia, head tremor, and reduced oculovestibular reflex activity (Figure 1), along with other pathophysiological characteristics like abnormal concentrations or decreases of certain substances (Podell et al., 2003; van Vliet et al., 2008; Choi et al., 2011; Lee et al., 2013; Yang Y. et al., 2020). However, despite their potential, the utilization of dogs in PD research remains relatively uncommon due to their high cost, ethical considerations and limited availability of genetically modified lines.

##### Non-human primates

Finally, non-human primate (NHP) models have played a crucial role in elucidating the pathophysiology of PD, as their genetic, physiological, and behavioral similarities to humans enable the reproduction of distinct disease stages that closely parallel those observed in patients (Blesa et al., 2018).

###### Non-human primate—toxic-based

The most extended toxic model of NHPs is the MPTP model. This MPTP model simulates the topographic pattern of dopaminergic degeneration in the striatum characteristic of PD (Blesa et al., 2012; Del Rey et al., 2024).

Depending on the toxic administration protocol, the acute and chronic stages of the pathological process can be reproduced (Figure 1; Blesa et al., 2011, 2012; Seo et al., 2019; Molinet-Dronda et al., 2022). However, the MPTP-NHP model has the same problem as other drug-induced models: the lack of *α*-syn aggregates (Halliday et al., 2009). Even so, this model has been essential in PD neuropharmacology to discern the side effects of L-DOPA and characterize the motor fluctuations induced by L-DOPA typical of patients (Cenci and Crossman, 2018; Hamadjida et al., 2020). In addition, in the pharmacological field, NHPs have tended to be used as a last step toward the clinic due to the advantages and disadvantages exposed above. Therefore, there are only a few drugs tested in this model. One example of the use of NHP could be trophic factors, administered intraparenchymal freely or in AAV. Mainly, toxic models have been used for research with trophic factors such as CDNF and GDNF, capable of promoting neuronal survival and repair (Garea-Rodríguez et al., 2016; Kordower and Burke, 2018). Such is the efficacy that several clinical trials tested these compounds (NCT01621581 and NCT06285643) (see Supplementary Table 1). One example is the effectiveness of AA2-GDNF, which is now in Phase II of a clinical trial after successful completion of Phase I (NCT06285643) (see Supplementary Table 1).

###### Non-human primate—genetic

Due to the absence of α-syn aggregates in the MPTP-NHP model, studies like that of Kirik et al. explored advancements in viral vector technology, enabling the unilateral overexpression of human wild-type α-syn and the A53T mutant α-syn in the SNc of adult marmosets (Kirik et al., 2003). Both wild-type and mutant *A53T* animals developed PD-like pathological manifestation (Figure 1) and histopathological studies revealed α-syn cytoplasmatic inclusions, granular deposits, and dystrophic neurites as well as 30–60% degeneration of DA neurons in the SNc (Kirik et al., 2003). However, subsequent studies using different viral vectors in NHPs showed variability in results between species (Eslamboli et al., 2007; Bourdenx et al., 2015; Yang et al., 2015; Marmion and Kordower, 2018). Precisely, Bourdenx et al. (2015) did not find any aging effects on the nigrostriatal pathway due to overexpression of mutant A53T α-syn via a recombinant AAV2/9 vector, which had previously been effective in rats. Recently, Koprich et al. (2016) conducted a series of experiments testing an AAV1/2 vector with overexpression of mutant A53T α-syn, which showed a 50% loss of nigral DA neurons and a 60% reduction in striatal DA content after 4 months (Koprich et al., 2016).

On the other hand, TG-NHPs are not abundant due to ethical concerns, cost and long gestation and maturation. However, the study of Niu et al. (2015) developed the first progressive transgenic model of PD. They used a lentiviral vector expressing A53T α-syn under human ubiquitin promoter in a rhesus macaque. This model showed an age-dependent non-motor symptoms such as cognitive impairments and some fine motor coordination deficits, though no clear evidence of neuronal degeneration in the magnetic resonance imaging (MRI) was observed (Niu et al., 2015).

In summary, vertebrate models provide a tiered framework that integrates increasing experimental complexity with enhanced translational relevance. Lower vertebrates like zebrafish enable rapid screening, and rodents offer a standardized stage. On the other hand, larger mammals support therapy development and pharmacokinetic testing, and NHPs provide the most clinically relevant models for evaluating safety and efficacy prior to human trials. A clear understanding of the strengths and limitations of each model is critical for aligning model selection with the specific research question and phase of drug development.

### Modeling non-motor symptoms of Parkinson’s disease

As outlined above, non-motor symptoms constitute a critical component of PD, emerging well before dopaminergic degeneration and underscoring the need for their early detection. Because of that, animal models focused on this aspect are essential to develop new therapeutical strategies to treat early symptoms such as: hyposmia, constipation, alteration in REM, RBD, anxiety/depression, and cognitive decline, as the most common ones in PD (Postuma et al., 2015; Heinzel et al., 2019).

Animal models for prodromal PD are mainly based on toxins, genetics, and overexpression of *α*-syn. It should also be noted that not all animal models described above can reproduce this phase of PD, because of either the type of species used or the poor reproducibility of the model (Taguchi et al., 2020). Furthermore, many non-motor symptoms of PD observed in animal models—as in patients—can arise from distinct underlying mechanisms, and their manifestation may vary considerably not only between different studies but also within the same model. This variability highlights the complexity of accurately replicating non-motor features of PD in preclinical research (Wichmann et al., 2025).

#### Invertebrate models

##### Drosophila melanogaster

Invertebrate models have also contributed to the study of certain non-motor symptoms-related mechanisms. In Drosophila, transgenic lines have shown certain life activities which depends on normal DA levels: as olfactory conditioning, sleep and arousal regulation, and memory and learning process (Figure 1; Selcho et al., 2009; Berry et al., 2012; Ueno et al., 2012; Dung and Thao, 2018). Specifically, PINK1 and parkin null Drosophila models have reported changes in learning and memory and in circadian rhythms (Julienne et al., 2017). Moreover, disruptions of light/dark behavior have been corroborated though electrophysiological studies in l-LNv clock neurons (responsible for circadian rhythms synchronization) which have shown an abnormal activity (Parisky et al., 2008; Hirsh et al., 2010; Shang et al., 2011; Julienne et al., 2017). The number of drugs tested in this non-motor model are few, however, Lawal et al., conducted unbiased Drosophila neuropsychiatric screens that identified aminergic agents and additional candidates affecting dopaminergic and sleep/circadian phenotypes, illustrating the platform’s capacity to uncover repurposing leads for PD (Lawal et al., 2014).

#### Vertebrate models

##### Zebrafish

Although less explored for non-motor symptoms, zebrafish models are increasingly used in this area. Zebrafish possess a pool of cognitive processes comprising learning, memory, fear, anxiety, perception, social skills and sleep patterns which can provide the opportunity of non-motor studies and the research of the prodromic phase of PD (Bailey et al., 2013). For example, rotenone-exposed adults display PD-like motor and non-motor phenotypes (Wang et al., 2017); cognitive readouts such as short-term avoidance memory and lateralization are robust (Figure 1; Fontana et al., 2018); and standardized adult pipelines allow concurrent quantification of motor and non-motor behaviors (Selvaraj et al., 2019). However, the absence of higher cortical structures and the limited repertoire of measurable affective behavior restrict the depth of non-motor symptoms modeling in this type of species.

##### Rodents

###### Rodent—toxic-based

In rodents, it has been demonstrated that olfactory dysfunction exists after the first injection with MPTP in a mouse model (Schintu et al., 2009), after intranasal administration of MPTP in rats, and also administering rotenone in rats and mice (Figure 1; Prediger et al., 2009; Babatunde et al., 2023; Yuan et al., 2023). Similarly, another of the non-motor symptoms studies from which results have been obtained in most traditional toxin models is constipation; MPTP exposure in mice has been reported to decrease the population of dopaminergic neurons and produce gastrointestinal impairments with an increment of intestinal motility and relaxation of the colon (Anderson and Gibbons, 2007). Also, rotenone-treated rats have been shown to show abnormal gastrointestinal function (Drolet et al., 2009). Moreover, rotenone-treated rats and in general MPTP-treated rodents shown sleep and circadian rhythm alterations (Laloux et al., 2008; Chen et al., 2013). Finally, in the 6-OHDA rat model, the reduction of dopaminergic neurons that is considered responsible for the RBD expressed in this model has been reported several times. However, there were no pathological changes in the most affected regions in RBD (Vo et al., 2014; Medeiros et al., 2019). In these cases, Ceftriaxone and intranasal insulin have been tested in MPTP rat models and 6-OHDA rat model demonstrating good effects in working memory, object recognition and cognitive deficits (Figure 1; Huang et al., 2015; Hsieh et al., 2017; Yang L. et al., 2020). Both drugs are ongoing in clinical trials with non-motor symptoms PD patients (NCT03413384 and NCT02064166) (see Supplementary Table 1; Degirmenci et al., 2023).

###### Rodent—genetic

Various strategies have been employed to generate different non-motor phenotypes in TG models. One example involves prion promoter-mice, where they exhibit reduced intestinal motility at 3 months, accompanied by the emergence of *α*-syn aggregates in the mesenteric and submucosal plexus of the colon (Rota et al., 2019; Taguchi et al., 2020). Another approach utilizes the Thy1-promoter, effectively replicating many non-motor symptoms in mice. This includes hyposmia at 3–5 months, anxiety at 4 months, sleep disturbances at 10 months, and intestinal dysfunction at 12 months (Fleming et al., 2008; Taylor et al., 2009; Chesselet et al., 2012; Taguchi et al., 2020). A third strategy employs endogenous α-syn promoters using bacterial artificial chromosome (BAC) and P1-derived artificial chromosome (PAC). A53T-SNCA BAC models have reported partial replication of the early PD process, encompassing non-motor symptoms like hyposmia and RBD (Taguchi et al., 2020). Conversely, A53T SNCA mice with PAC demonstrated reduced intestinal motility and prolonged intestinal transit time (Kuo et al., 2010). Rasagiline in this case has shown improvements in olfactory deficits in an α-syn-mouse model, overexpressing wild-type human α-syn (Petit et al., 2013), and has been tested in PD patients with different non-motor symptoms as hyposmia, apathy, cognition or sleep (NCT00902941, NCT00755027, NCT01382342, NCT01032486, respectively) (see Supplementary Table 1). Another example, is Buntanetap (also known as posiphen or ANVS-40), an enantiomer traditionally inhibitor that reduces amyloid precursor protein and α-synuclein expression in brain and gut of A53T transgenic mice (Kuo et al., 2019). The clinical trials were completed in December of 2021 with positive results in terms of improvements of motor function (especially in patients with a longer disease duration) and a maintenance or improvement in cognitive state in certain subgroups, with an acceptable safety and tolerability profile, and side effects mainly attributed to CSF catheterization for the SILK analysis (NCT05357989); full peer-reviewed efficacy results are pending (see Supplementary Table 1; Galasko et al., 2024).

Mitochondrial models such as Mitopark mice, in which the mitochondrial transcription factor TFAM in specifically deleted (Westerlund et al., 2008), have described in the studies certain non-motor deficits; circadian desynchronization, early cognitive impairments (including olfactory disfunction, learning and memory deficits), anxiety or depression (Figure 1; Li et al., 2013; Fifel and Cooper, 2014; Langley et al., 2021).

Finally, it is important to consider the route of administration when modeling α-synucleinopathies and non-motor features in mice. Injections of α-syn fibrils into the olfactory bulb or the gastrointestinal tract reproduce non-motor symptoms alongside α-syn pathology. By contrast, subcutaneous administration has been reported to elicit non-motor readouts without robust central α-syn pathology. A recent alternative is intravenous delivery using viral vectors: in addition to inducing α-syn pathology across multiple brain regions and the small intestine, this approach yields measurable non-motor phenotypes—for example, olfactory-bulb seeding drives pathology within olfactory circuits with early neuronal loss; gastrointestinal inoculation produces enteric α-syn pathology with impaired gastrointestinal transit; and intravenous AAV-mediated α-syn overexpression leads to widespread central and enteric pathology accompanied by anxiety-like behavior and mild deficits in attention/working-memory paradigms (Manfredsson et al., 2018; Del Rey N. L. et al., 2018; Kuan et al., 2021).

##### Dogs

Interestingly, dog gut microbiome more closely resembles the human gut microbiome in composition and functional overlap, compared with rodent models. Therefore, it may be a potential model for the study of gut-brain interactions in PD. However, the canine models represent a minority in case of PD research (Ambrosini et al., 2019).

##### Non-human primates

###### Non-human primate—toxic-based

The MPTP-NHP model has revealed loss of REM sleep and increased napping after the first intravenous injection of MPTP (0.5 mg/kg) (Barraud et al., 2009). Following studies have monitored that electroencephalographic activity shows suppression of REM sleep after first MPTP injection and chronic MPTP administration results in progressive sleep deterioration, fragmentation, and reduced sleep efficacy with a corresponding increased sleepiness during the day by about 50% (Barraud et al., 2009; Fox and Brotchie, 2010). Moreover, studies in MPTP marmosets have reported an increased muscle tone during REM sleep (Fox and Brotchie, 2010; Verhave et al., 2011; Bezard and Fernagut, 2014; Bao et al., 2023). Additionally, MPTP-NHP model has also been used to describe cognitive deficits during attentional and executive task performance, by measuring visual discrimination, delayed response or matching-to-sample, object retrieval/detour tasks that are impaired even in MPTP-treated NHPs with minimal motor deficits (Figure 1; Bao et al., 2023). Furthermore, assessments of self-initiated and visually-triggered saccades in MPTP monkeys have revealed increased errors, including an increased tendency to initiate inappropriate or premature responses (Schneider and Kovelowski, 1990; Taylor et al., 1990; Slovin et al., 1999; Pessiglione et al., 2008; Fox and Brotchie, 2010). A good example of preclinical study in MPTP models is the NYX-458, a NMDAR modulator which improves cognitive performance in terms of working memory, attention and executive function (Barth et al., 2020; Degirmenci et al., 2023). NYX-458 effects has been demonstrating in an active Phase II clinical trial on mild cognitive impairment, mild dementia associated with PD or prodromal or manifest Lewy Body dementia (NCT04148391) (see Supplementary Table 1). Indeed, the MPTP-NHP model stands as the gold standard when it comes to study cognitive and sleep deficits in PD. This model has proven to be invaluable in enhancing outcome measurements, facilitating the translation of preclinical findings into potentially beneficial drug interventions for PD.

In summary, although modeling non-motor symptoms remains a challenge due to their heterogeneity and complexity, significant progress has been made across species. Rodents and NHPs currently allow the most robust approaches to assessing cognitive, olfactory, and gastrointestinal symptoms of PD, while invertebrates and fish models provide understanding of individual physiological processes. A comprehensive understanding of non-motor symptoms requires integrative approaches that combine behavioral, histological, and molecular strategies in different models.

## Conclusion

**Figure 2:**
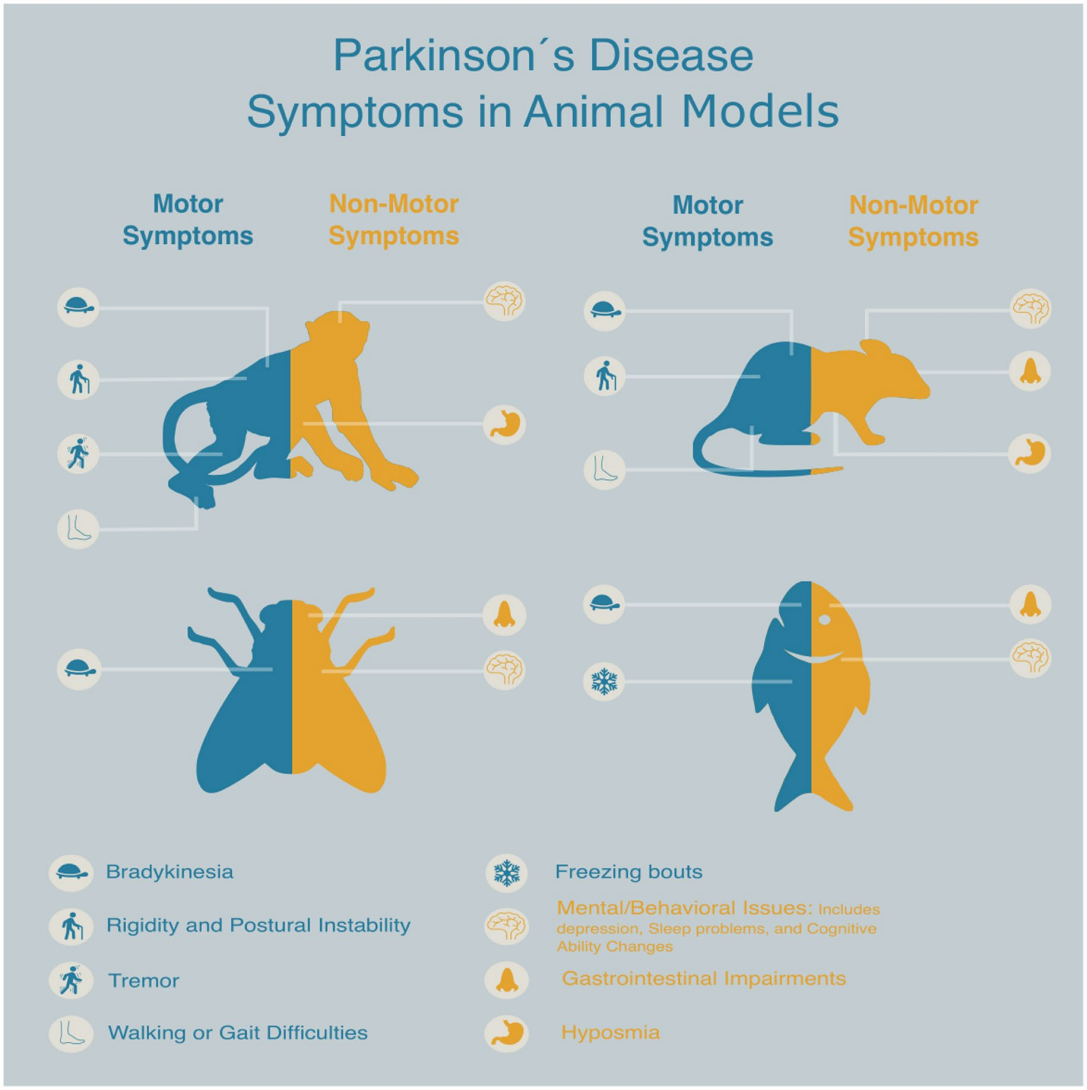
Schematic representation of key animal models commonly used in Parkinson’s disease drug discovery, highlighting the presence and treatment of both motor and non-motor symptoms associated with each model.

Animal models are indispensable tools for investigating the complex pathophysiology of PD and for evaluating novel therapeutic strategies. They have significantly advanced our understanding of the disease and facilitated the identification of promising new compounds. Nevertheless, despite decades of research with a broad spectrum of preclinical models—from cellular models and rodent platforms to NHPs—PD still lacks a disease modifying therapy. This unresolved challenge underscores a critical gap: while current models reproduce many motor and non-motor features of PD, none of them fully capture its complexity, heterogeneity, or progressive nature of the disorder (Figure 2).

Each model contributes to our understanding of PD pathophysiology, yet none is sufficient on its own. A clear example of this limitation is the repeated failure of many promising compounds that perform well in preclinical stages but do not translate into successful clinical outcomes, highlighting the restricted predictive validity of existing systems. Cellular and invertebrate models enable mechanistic studies and pharmacological screening rodent models provide reproducible platforms for behavioral or molecular analyses; then NHP models offer high translational value for testing therapeutic strategies. Moreover, most models have traditionally focused on motor deficits, although by the time these appear neurodegeneration has already begun; this realization has shifted attention toward non-motor symptoms and prodromal stages as earlier and more predictive indicators of the disease (Table 1).

**Table 1:** Summary of experimental models commonly employed in Parkinson’s disease (PD) research.

Model	Key strengths	Key limitations
*In vitro*	Human-relevant; patient-specific genetics; scalable HTS; α-syn assays/organoid 3D context	No vasculature/immune cells; batch variability; limited systems-level translation
Invertebrates	Ultra-fast genetics; low cost; large screens; conserved pathways	No myelin/BBB; simple behavior; anatomical divergence
Zebrafish	Vertebrate; optical access; easy drug delivery; medium throughput	Early-life/acute phenotypes; brain & regeneration differences; limited complex behavior
Rodents	Mammalian CNS; rich motor/non-motor battery; progressive setups possible; α-syn propagation (AAV/PFF); mitochondrial dysfunction models.	Species differences; many genetic lines with modest DA loss; toxin models often acute; systemic/aging-like, PD specificity limited
Large mammals	Brain size/connectivity > rodents; translational delivery/testing	High cost/logistics; fewer genetic tools/lines; smaller literature base
Non-human primates	Closest to humans; robust motor phenotypes; translational/biomarker/device readouts	Very high cost/ethics; small n; MPTP lacks Lewy pathology

The table highlights key strengths, including genetic tractability, scalability, and translational relevance, as well as major limitations such as species-specific anatomical and physiological differences, lack of full disease pathology (e.g., Lewy bodies), and constraints related to cost, ethics, or experimental feasibility.

Looking ahead, improving the predictive power of PD models will require an integrative approach that incorporates genetic, environmental, and age-related factors reflecting both motor and non-motor aspects across disease progression. Greater alignment between preclinical endpoints and clinical outcomes, together with stronger emphasis on inter-individual variability, will also be a key to boosting translational applicability. Ultimately, the selection and refinement of existing models, alongside the development of new and more representative platforms, will be key to more accurately evaluating novel therapies, identifying reliable biomarkers for early intervention, and effectively bridging the gap between promising preclinical findings and meaningful clinical benefits for patients with PD.
