# Supplementary material for: Drug discovery and development for Parkinson’s disease: are preclinical models good enough?
Source: Front Aging Neurosci. 2025 Oct 28;17:1692592. doi: 10.3389/fnagi.2025.1692592 (PMC12602412; doi:10.3389/fnagi.2025.1692592)
Supplement: Supplementary file 1 [file Data_Sheet_1.pdf]

| *Candidate* | *Clinical domain* | *Clinical status / outcome* | *Trial* | *Preclinical model used* | *Reference* |
|---|---|---|---|---|---|
| *AB-1005* | Motor | Phase 1 completed; Phase 1b safety; Phase 2 on-going | NCT01621581; NCT04167540; NCT06285643 | NHP–MPTP; Rodent toxins | Eberling *et al.*, 2009; Kells *et al.*, 2010 |
| *ABBV-0805* | Motor | Phase 1 completed | NCT04127695 | Rodent α-syn TG/PFF | Nordström *et al.*, 2021 |
| *Ambroxol* | Motor & Non-motor | Phase 2 on-going; Phase 2 completed | NCT02914366; NCT02941822 | Rodent α-syn/GBA models | Migdalska-Richards *et al.*, 2016, 2017 |
| *Buntanetap/ANVS401 (posiphen)* | Motor | Phase 1-2 completed; Phase 3 completed | NCT04524351; NCT05357989 | A53T α-syn TG mouse; α-syn model | Kuo *et al.*, 2019 |
| *CDNF* | Motor | Phase 1/2 completed | NCT03295786; NCT03775538 | 6-OHDA rat; MPTP mouse; NHP–6-OHDA | Garea-Rodríguez *et al.*, 2016 |
| *Ceftriaxone* | Non-motor | Phase 2 on-going | NCT03413384 | MPTP rat; MPTP mouse | Bisht *et al.*, 2014; Zhou *et al.*, 2021 |
| *Exenatide (GLP-1RA)* | Motor | Phase 3 on-going | NCT04232969 | MPTP mouse; 6-OHDA rat; MitoPark | Kim, Moon and Park, 2009; Kalinderi, Papaliagkas and Fidani, 2024 |
| *HE3286 (androstenetriol)* | Motor | Phase 2 completed | NCT05083260 | MPTP mouse | Nicoletti *et al.*, 2012 |
| *Hypoestoxide* | Motor | Phase 2 completed | NCT04858074 | Rodent α-syn TG | Kim *et al.*, 2015 |
| *Intranasal insulin* | Non-motor | Phase 2 completed; | NCT02064166; NCT02577523; NCT04687878 | 6-OHDA rat | Pang *et al.*, 2016; Fine *et al.*, 2020; Yang *et al.*, 2020 |
| *NYX-458* | Non-motor | Phase 2 on-going | NCT04148391 | NHP–MPTP | Barth *et al.*, 2020 |
| *Nilotinib — check citation* | Motor | Phase 2 completed | NCT02954978 | Rodent α-syn/c-Abl | Hebron, Lonskaya and Moussa, 2013; Karuppagounder *et al.*, 2014 |

| | | | | | |
|---|---|---|---|---|---|
| *Rasagiline (MAO-B inhibitor)* | Non-motor | Phase 2 completed; Phase 4 completed; Oobservational; Phase 4 completed; Phase 2 completed | NCT00755027; NCT00902941; NCT01032486; NCT01382342; NCT02789020 | α-syn TG mouse; DA lesion models; Zebrafish MPTP | Petit *et al.*, 2013; Cronin and Grealy, 2017 |
| *Tozadenant* | Motor | Phase 3 terminated; Phase 1-2 completed | NCT02453386; NCT04524351 | 6-OHDA rodent; NHP–MPTP | Michel *et al.*, 2015, 2017 |
| *UB-312* | Motor | Phase 1 completed | NCT04075318 | Rodent α-syn TG/PFF | Nimmo *et al.*, 2022 |
| *UCB0599 / minzasolmin* | Motor | Phase 2 completed; Phase 1 completed; Phase 2 terminated | NCT04658186; NCT04875962; NCT05543252 | Rodent α-syn TG/PFF | Price *et al.*, 2018, 2023 |
| *Vipadenant* | Motor | Phase 2 completed; | NCT00438607; NCT00442780 | 6-OHDA rodent; NHP–MPTP | Kanda and Jenner, 2020 |
| *Prasinezumab / PRX002* | Motor | Phase 2 on-going; Phase 2 missed primary | NCT03100149; NCT04777331 | Rodent α-syn TG & PFF | Schenk *et al.*, 2017; Pagano *et al.*, 2021 |
| *Preladenant* | Motor | Phase 3 Terminated | NCT01155466; NCT01155479 | 6-OHDA rodent; NHP–MPTP | Kanda and Jenner, 2020 |
| *Istradefylline* | Motor | FDA approved | NCT00456794 | 6-OHDA rat; NHP–MPTP | Kadowaki Horita *et al.*, 2013; Ko *et al.*, 2016 |
| *Lixisenatide* | Motor | Phase 2 completed | NCT03439943 | MPTP mouse | Liu *et al.*, 2015 |
| *ND0612* | Motor | Phase 2 completed; Phase 2 on-going | NCT02577523; NCT02726386 | Large animal PK/toxicology (minipig/dog) | Ramot *et al.*, 2017 |
| *NLY01 (PEG-exenatide)* | Motor | Phase 2 completed | NCT04154072 | Rodent toxin | Kim, Moon and Park, 2009 |
| *Cinpanemab / BIIB054* | Motor | Phase 2 terminated | NCT03318523 | Rodent α-syn PFF; α-syn TG | Weihofen *et al.*, 2019 |

| | | | | | |
|---|---|---|---|---|---|
| *Coenzyme Q10* | Motor | Phase 3 terminated | NCT00740714; | MPTP mouse; rotenone rat; *C. elegans* | Beal *et al.*, 1998; Yang *et al.*, 2009 |
| *Creatine* | Motor | Phase 3 terminated | NCT00449865 | MPTP mouse; 6-OHDA rat; cell models | Matthews *et al.*, 1999; Yang *et al.*, 2009 |

## References


Barth, A.L. *et al.* (2020) "NYX-458 Improves Cognitive Performance in a Primate Parkinson's Disease Model," *Movement Disorders*, 35(4), pp. 640–649. Available at: https://doi.org/10.1002/mds.27962.

Beal, M.F. *et al.* (1998) "Coenzyme Q10 attenuates the 1-methyl-4-phenyl-1,2,3,6-tetrahydropyridine (MPTP) induced loss of striatal dopamine and dopaminergic axons in aged mice," *Brain Research*, 783(1), pp. 109–114. Available at: https://doi.org/10.1016/S0006-8993(97)01192-X.

Bisht, R. *et al.* (2014) "Ceftriaxone mediated rescue of nigral oxidative damage and motor deficits in MPTP model of Parkinson's disease in rats," *NeuroToxicology*, 44, pp. 71–79. Available at: https://doi.org/10.1016/j.neuro.2014.05.009.

Cronin, A. and Grealy, M. (2017) "Neuroprotective and Neuro-restorative Effects of Minocycline and Rasagiline in a Zebrafish 6-Hydroxydopamine Model of Parkinson's Disease," *Neuroscience*, 367, pp. 34–46. Available at: https://doi.org/10.1016/j.neuroscience.2017.10.018.

Eberling, J.L. *et al.* (2009) "Functional Effects of AAV2-GDNF on the Dopaminergic Nigrostriatal Pathway in Parkinsonian Rhesus Monkeys," *Human Gene Therapy*, 20(5), pp. 511–518. Available at: https://doi.org/10.1089/hum.2008.201.

Fine, J.M. *et al.* (2020) "Intranasal delivery of low-dose insulin ameliorates motor dysfunction and dopaminergic cell death in a 6-OHDA rat model of Parkinson's Disease," *Neuroscience Letters*, 714, p. 134567. Available at: https://doi.org/10.1016/j.neulet.2019.134567.

Garea-Rodríguez, E. *et al.* (2016) “Comparative Analysis of the Effects of Neurotrophic Factors CDNF and GDNF in a Nonhuman Primate Model of Parkinson’s Disease,” *PloS One*, 11(2), p. e0149776. Available at: https://doi.org/10.1371/journal.pone.0149776.

Hebron, M.L., Lonskaya, I. and Moussa, C.E.-H. (2013) “Nilotinib reverses loss of dopamine neurons and improves motor behavior via autophagic degradation of α-synuclein in Parkinson’s disease models,” *Human Molecular Genetics*, 22(16), pp. 3315–3328. Available at: https://doi.org/10.1093/hmg/ddt192.

Kadowaki Horita, T. *et al.* (2013) “Effects of the adenosine A2A antagonist istradefylline on cognitive performance in rats with a 6-OHDA lesion in prefrontal cortex,” *Psychopharmacology*, 230(3), pp. 345–352. Available at: https://doi.org/10.1007/s00213-013-3158-x.

Kalinderi, K., Papaliagkas, V. and Fidani, L. (2024) “GLP-1 Receptor Agonists: A New Treatment in Parkinson’s Disease,” *International Journal of Molecular Sciences*, 25(7). Available at: https://doi.org/10.3390/ijms25073812.

Kanda, T. and Jenner, P. (2020) “Can adenosine A2A receptor antagonists modify motor behavior and dyskinesia in experimental models of Parkinson’s disease?,” *Parkinsonism & Related Disorders*, 80, pp. S21–S27. Available at: https://doi.org/10.1016/j.parkreldis.2020.09.026.

Karuppagounder, S.S. *et al.* (2014) “The c-Abl inhibitor, Nilotinib, protects dopaminergic neurons in a preclinical animal model of Parkinson’s disease,” *Scientific Reports*, 4(1), p. 4874. Available at: https://doi.org/10.1038/srep04874.

Kells, A.P. *et al.* (2010) “Regeneration of the MPTP-lesioned dopaminergic system after convection-enhanced delivery of AAV2-GDNF,” *The Journal of Neuroscience: The Official Journal of the Society for Neuroscience*, 30(28), pp. 9567–9577. Available at: https://doi.org/10.1523/JNEUROSCI.0942-10.2010.

Kim, C. *et al.* (2015) “Hypoestoxide reduces neuroinflammation and α-synuclein accumulation in a mouse model of Parkinson’s disease,” *Journal of Neuroinflammation*, 12(1), p. 236. Available at: https://doi.org/10.1186/s12974-015-0455-9.

Kim, S., Moon, M. and Park, S. (2009) “Exendin-4 protects dopaminergic neurons by inhibition of microglial activation and matrix metalloproteinase-3 expression in an animal model of Parkinson’s disease,” *The Journal of Endocrinology*, 202(3), pp. 431–439. Available at: https://doi.org/10.1677/JOE-09-0132.

Ko, W.K.D. *et al.* (2016) “An evaluation of istradefylline treatment on Parkinsonian motor and cognitive deficits in 1-methyl-4-phenyl-1,2,3,6-tetrahydropyridine (MPTP)-treated macaque models,” *Neuropharmacology*, 110(Pt A), pp. 48–58. Available at: https://doi.org/10.1016/j.neuropharm.2016.07.012.

Kuo, Y.-M. *et al.* (2019) “Translational inhibition of α-synuclein by Posiphen normalizes distal colon motility in transgenic Parkinson mice,” *American Journal of Neurodegenerative Disease*, 8(1), pp. 1–15.

Liu, W. *et al.* (2015) “Neuroprotective effects of lixisenatide and liraglutide in the 1-methyl-4-phenyl-1,2,3,6-tetrahydropyridine mouse model of Parkinson’s disease,” *Neuroscience*, 303, pp. 42–50. Available at: https://doi.org/10.1016/j.neuroscience.2015.06.054.

Matthews, R.T. *et al.* (1999) “Creatine and Cyclocreatine Attenuate MPTP Neurotoxicity,” *Experimental Neurology*, 157(1), pp. 142–149. Available at: https://doi.org/10.1006/exnr.1999.7049.

Michel, A. *et al.* (2015) “Behavioural Assessment of the A2a/NR2B Combination in the Unilateral 6-OHDA-Lesioned Rat Model: A New Method to Examine the Therapeutic Potential of Non-Dopaminergic Drugs,” *PLOS ONE*, 10(8), p. e0135949. Available at: https://doi.org/10.1371/journal.pone.0135949.

Michel, A. *et al.* (2017) “Antiparkinsonian effects of the ‘Radiprodil and Tozadenant’ combination in MPTP-treated marmosets,” *PloS One*, 12(8), p. e0182887. Available at: https://doi.org/10.1371/journal.pone.0182887.

Migdalska-Richards, A. *et al.* (2016) “Ambroxol effects in glucocerebrosidase and α-synuclein transgenic mice,” *Annals of Neurology*, 80(5), pp. 766–775. Available at: https://doi.org/10.1002/ana.24790.

Migdalska-Richards, A. *et al.* (2017) “Oral ambroxol increases brain glucocerebrosidase activity in a nonhuman primate,” *Synapse (New York, N.Y.)*, 71(7), p. e21967. Available at: https://doi.org/10.1002/syn.21967.

Nicoletti, F. *et al.* (2012) “17α-Ethynyl-androst-5-ene-3β,7β,17β-triol (HE3286) Is Neuroprotective and Reduces Motor Impairment and Neuroinflammation in a Murine MPTP Model of Parkinson’s Disease,” *Parkinson’s Disease*, 2012(1), p. 969418. Available at: https://doi.org/10.1155/2012/969418.

Nimmo, J.T. *et al.* (2022) “Immunisation with UB-312 in the Thy1SNCA mouse prevents motor performance deficits and oligomeric α-synuclein accumulation in the brain and gut,” *Acta Neuropathologica*, 143(1), pp. 55–73. Available at: https://doi.org/10.1007/s00401-021-02381-5.

Nordström, E. *et al.* (2021) “ABBV-0805, a novel antibody selective for soluble aggregated α-synuclein, prolongs lifespan and prevents buildup of α-synuclein pathology in mouse models of Parkinson’s disease,” *Neurobiology of Disease*, 161, p. 105543. Available at: https://doi.org/10.1016/j.nbd.2021.105543.

Pagano, G. *et al.* (2021) “A Phase II Study to Evaluate the Safety and Efficacy of Prasinezumab in Early Parkinson’s Disease (PASADENA): Rationale, Design, and Baseline Data,” *Frontiers in Neurology*, 12, p. 705407. Available at: https://doi.org/10.3389/fneur.2021.705407.

Pang, Y. *et al.* (2016) “Intranasal insulin protects against substantia nigra dopaminergic neuronal loss and alleviates motor deficits induced by 6-OHDA in rats,” *Neuroscience*, 318, pp. 157–165. Available at: https://doi.org/10.1016/j.neuroscience.2016.01.020.

Petit, G.H. *et al.* (2013) “Rasagiline Ameliorates Olfactory Deficits in an Alpha-Synuclein Mouse Model of Parkinson’s Disease,” *PLOS ONE*, 8(4), pp. 1–13. Available at: https://doi.org/10.1371/journal.pone.0060691.

Price, D.L. *et al.* (2018) “The small molecule alpha-synuclein misfolding inhibitor, NPT200-11, produces multiple benefits in an animal model of Parkinson’s disease,” *Scientific Reports*, 8, p. 16165. Available at: https://doi.org/10.1038/s41598-018-34490-9.

Price, D.L. *et al.* (2023) “In vivo effects of the alpha-synuclein misfolding inhibitor minzasolmin supports clinical development in Parkinson’s disease,” *npj Parkinson’s Disease*, 9(1), p. 114. Available at: https://doi.org/10.1038/s41531-023-00552-7.

Ramot, Y. *et al.* (2017) “Ninety-day Local Tolerability and Toxicity Study of ND0612, a Novel Formulation of Levodopa/Carbidopa, Administered by Subcutaneous Continuous Infusion in Minipigs.,” *Toxicologic pathology*, 45(6), pp. 764–773. Available at: https://doi.org/10.1177/0192623317729891.

Schenk, D.B. *et al.* (2017) “First-in-human assessment of PRX002, an anti–α-synuclein monoclonal antibody, in healthy volunteers,” *Movement Disorders*, 32(2), pp. 211–218. Available at: https://doi.org/10.1002/mds.26878.

Weihofen, A. *et al.* (2019) “Development of an aggregate-selective, human-derived α-synuclein antibody BIIB054 that ameliorates disease phenotypes in Parkinson’s disease models,” *Neurobiology of Disease*, 124, pp. 276–288. Available at: https://doi.org/10.1016/j.nbd.2018.10.016.

Yang, L. *et al.* (2009) “Combination therapy with Coenzyme Q10 and creatine produces additive neuroprotective effects in models of Parkinson’s and Huntington’s Diseases,” *Journal of Neurochemistry*, 109(5), pp. 1427–1439. Available at: https://doi.org/10.1111/j.1471-4159.2009.06074.x.

Yang, L. *et al.* (2020) “Intranasal insulin ameliorates cognitive impairment in a rat model of Parkinson’s disease through Akt/GSK3β signaling pathway,” *Life Sciences*, 259, p. 118159. Available at: https://doi.org/10.1016/j.lfs.2020.118159.

Zhou, X. *et al.* (2021) “Neuroprotective Effect of Ceftriaxone on MPTP-Induced Parkinson’s Disease Mouse Model by Regulating Inflammation and Intestinal Microbiota,” *Oxidative Medicine and Cellular Longevity*, 2021(1), p. 9424582. Available at: https://doi.org/10.1155/2021/9424582.
